# Subacute Sclerosing Panencephalitis manifesting as Bell’s palsy and bilateral macular necrotizing retinitis: an atypical presenting feature

**DOI:** 10.1186/s12348-020-00223-1

**Published:** 2021-02-01

**Authors:** Lagan Paul, Tanya Jain, Manisha Agarwal, Shalini Singh

**Affiliations:** Vitreo-retina Department, Dr. Shroff’s Charity Eye Hospital, 5027, Kedarnath Road, Daryaganj, New Delhi, 110002 India

## Abstract

**Background:**

Subacute sclerosing panencephalitis (SSPE) is a potentially lethal complication of measles infection. Neurological complications take years to manifest after primary viral infection of brain and can lead to blindness in some individuals.

**Findings:**

A 13-year-old female patient with history of Bell’s palsy 2 months prior, presented with rapidly progressing necrotizing retinitis in both eyes. Soon after, she was unable to walk, developed myoclonic jerks, altered sensorium and loss of bowel and bladder control. Her clinical history, CSF IgG measles antibody analysis, MRI brain and EEG findings confirmed the diagnosis of SSPE.

**Conclusion:**

SSPE in our case presented as Bell’s palsy and sudden painless diminution of vision due to ocular involvement, and developed full blown disease within 2 months. SSPE can present as a diagnostic challenge and warrants early identification and referral for timely diagnosis and management.

## Introduction

Subacute sclerosing panencephalitis (SSPE) is a progressive neurodegenerative disease caused by persistence of defective measles infection commonly seen in children and young adults. It usually occurs 7–10 years after measles infection [1]. Ocular manifestations usually precede the neurological manifestations of the disease [1]. Our case presented with bell’s palsy as the first neurological sign and retinal involvement happened after 2 months. This case highlights the important role of an ophthalmologist in the diagnosis of this fatal disease and aiding the neurologist in timely intervention and proper rehabilitation.

## Case report

**Fig. 1:**
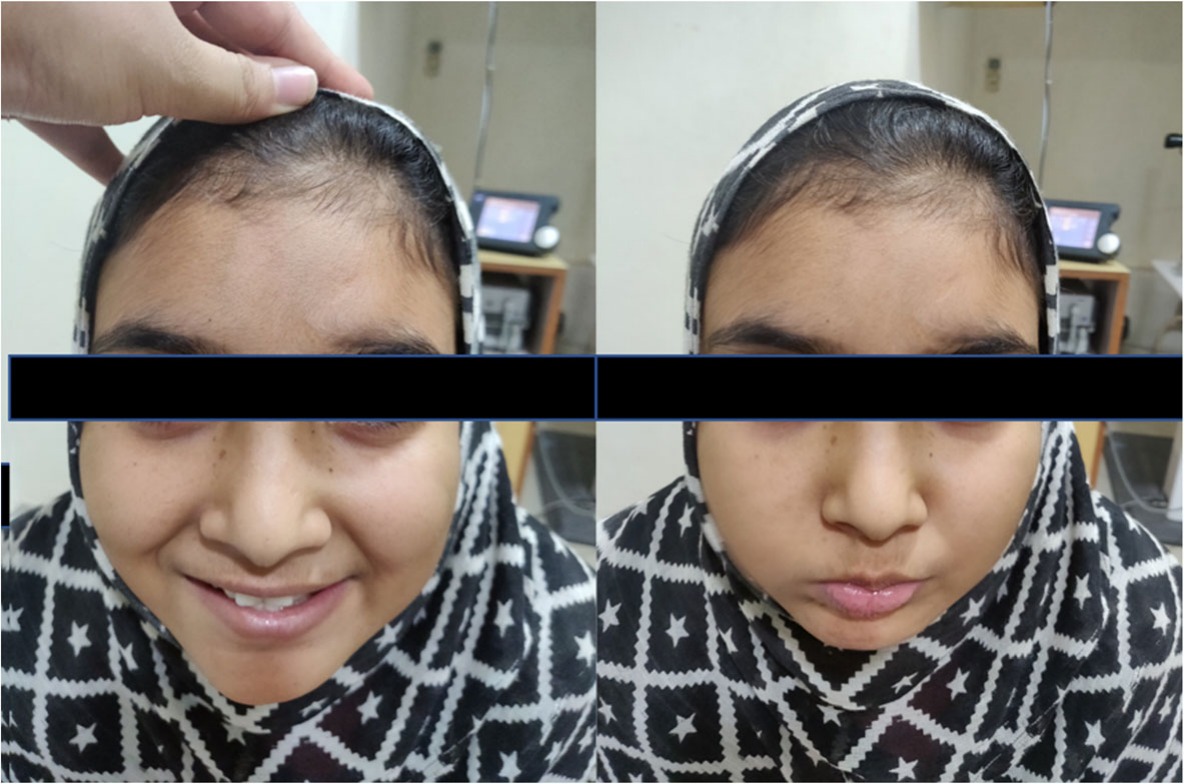
Photograph of patient showing residual facial palsy(deviation of angle of moth and decreased action of buccinator muscle supplied by facial nerve)

**Fig. 2:**
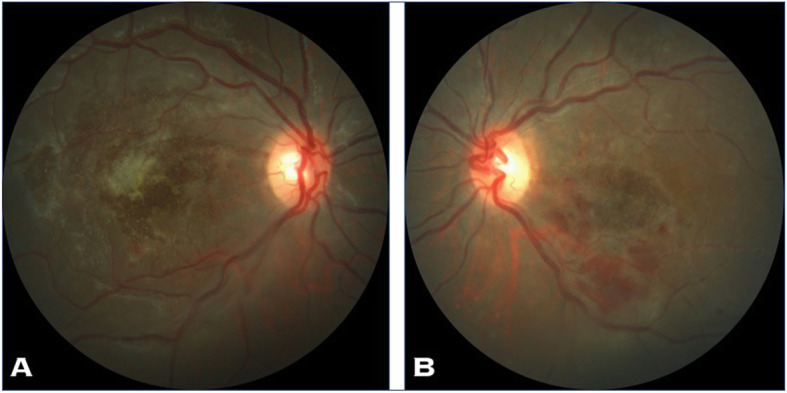
**a** and **b** Fundus photo of the patient at first visit showing macular necrotising retinitis

**Fig. 3:**
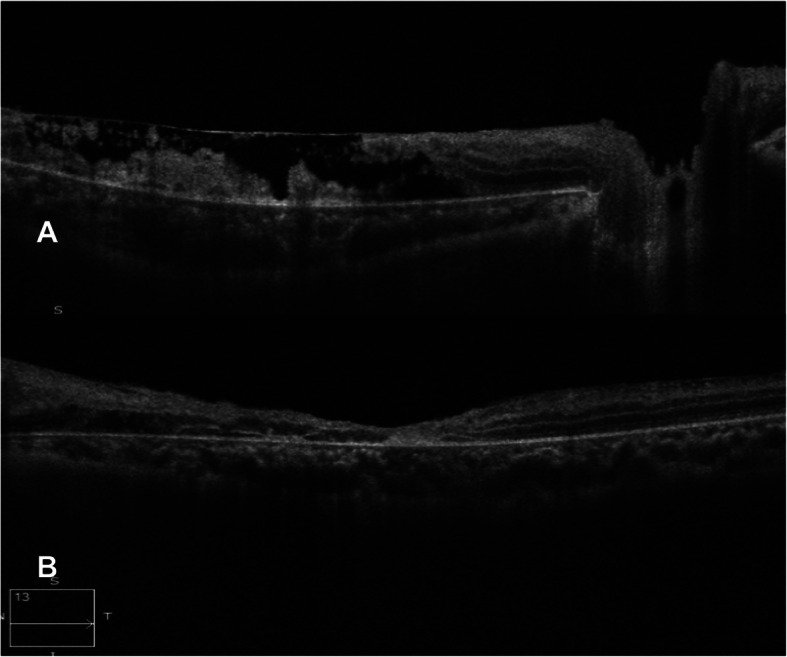
**a** SD-OCT of the RE showing loss of retinal architecture with moth eaten appearance of the retina. **b** SD-OCT of the LE showing loss of retinal architecture with foveal thinning

**Fig. 4:**
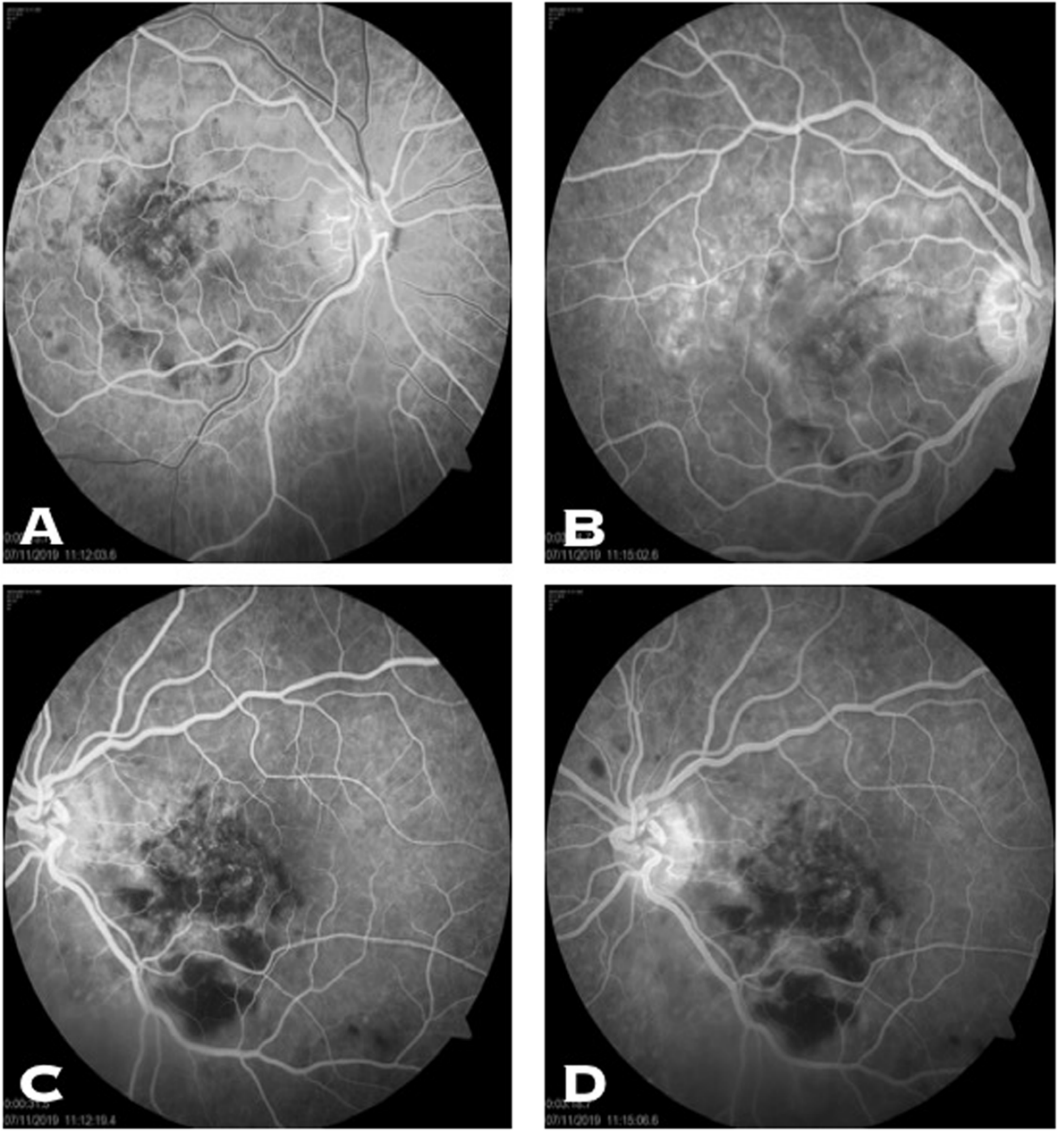
**a** and **b** FA of the RE showing early blockage of background fluorescence in area of active lesion with irregular staining corresponding to RPE atrophy, followed by late staining of the retinitis lesions. **c** and **d** FA of LE showed patchy hyperfluorecence corresponding to RPE atrophic patches with blocked fluorescence corresponding to intraretinal haemorrhages

A 13-year-old Asian Indian female presented to our clinic with complaint of diminution of vision in both eyes for the last 20 days. There was a history of acute onset left sided facial palsy 2 months back, which was treated with oral acyclovir but no neurological workup was performed then. Systemic history was unremarkable. She had no history of similar episode in the past and her immunisation status was not known. Central nervous system (CNS) examination was within normal limits apart from mild residual sequelae of facial palsy (Fig. 1). Her best corrected visual acuity (BCVA) in right eye (RE) was 1/60, Near <N/60 and 3/60, Near<N/36 in the left eye (LE). There was no evidence of anterior segment or vitreous inflammation. Her fundus examination revealed whitish ground glass retinitis lesions with ill-defined edges over the posterior pole with haemorrhages around it in RE. (Fig. 2) LE showed an area of thinning with RPE mottling and haemorrhages in macular area with no active retinal lesions. There was presence of associated mild venous dilation and areas of vascular occlusion and few healed lesions in mid-peripheral location. Optic nerve at presentation was normal in appearance and appeared healthy. Spectral domain OCT (SD-OCT) of RE showed loss of nerve fibre layer (NFL), ganglion cell layer (GCL) and inner and outer nuclear layers. Outer retinal appeared completely distorted and thinned out with intermittent complete loss of retinal tissue giving a moth-eaten appearance (Fig. 3a). OCT of LE revealed foveal thinning with total loss of retinal layer architecture.(Fig. 3b) Fluorescein angiogram (FA) of RE showed early blockage of background fluorescence in area of active white lesion with irregular staining corresponding to RPE atrophy, followed by late staining of the retinitis lesions (Fig. 4a, b). FA of LE showed patchy hyperfluorecence corresponding to RPE atrophic patches with blocked fluorescence corresponding to intraretinal haemorrhages (Fig. 4c, d). There was no associated disc or vessel staining in either eye.

**Fig. 5:**
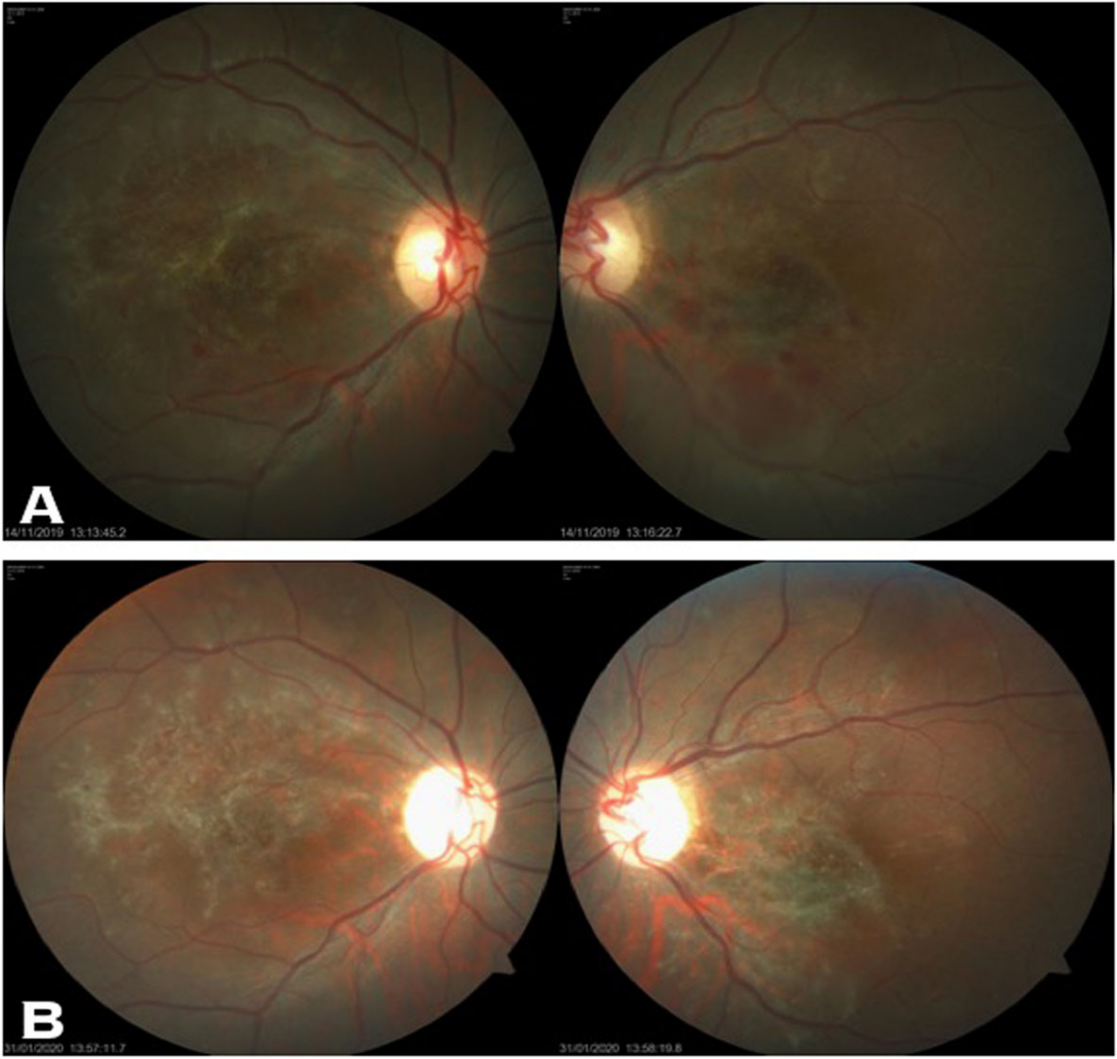
**a** Fundus photograph taken 10 days after commencement of treatment with oral steroids and oral antivirals showing healing retinitis patches. **b** Fundus photography taken after 2 months showing disc palor in both eyes and scarred retinitis patches

**Fig. 6:**
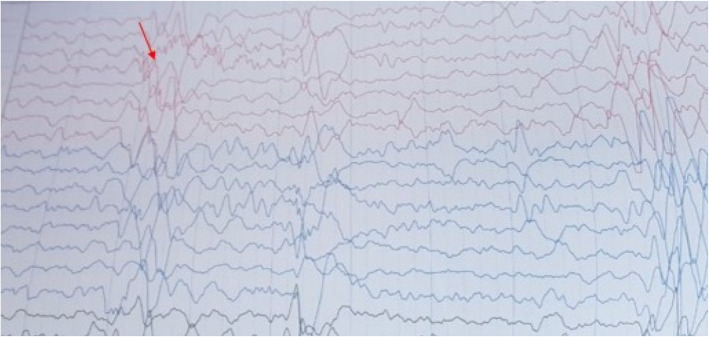
EEG report examination revealed intermittent high voltage slow wave discharges (red arrow)

Her systemic investigations were essentially within normal limits apart from lymphocytosis. Based on the clinical picture and investigations, a working diagnosis of viral retinitis was made and the patient was started on oral acyclovir (400 mg BD) and oral steroids at 1 mg/kg body weight in weekly tapering doses. On 10 days follow-up, the BCVA was same as before and the lesions appeared to be healing (Fig. 5a). However, due to a peculiar history of facial palsy, unknown immunisation status and macular necrotising retinitis, SSPE associated viral retinitis was suspected and the patient was referred to the paediatrician for further evaluation. While her ocular findings were concerning, there was a lack of systemic symptoms and neurological signs. Nevertheless, CSF examination and electroencephalography (EEG) may have been supportive of the diagnosis of SSPE at this time. However, the paediatrician opted for observation without at admission to the hospital.” Her magnetic resonance imaging (MRI) of the brain at this time was normal. We continued to follow the patient closely and after 2 months, the patient presented again with history of total loss of vision in both eyes with involuntary jerky movement of the right upper limb, generalised loss of sensorium and decreased awareness along with loss of bladder and bowel control since 10 days duration. The child was conscious, but not oriented and uncooperative for examination. On ocular examination, there was complete loss of light perception in both eyes with restricted ocular movements in all directions. Pupils were sluggishly reactive in both eyes and fundus examination revealed total disc pallor and healed retinitis lesions in the both eyes (Fig. 5b). A referral to a neurologist revealed that higher mental functions, cranial nerves, motor and sensory examination could not be performed as the patient was uncooperative for examination. Muscle strength was 3/5 and, while superficial and deep tendon reflexes could be elicited, the plantar reflex was equivocal or decreased. There was involuntary loss of bladder and bowel control. CSF analysis was done which revealed an opening pressure of 8 cm of water, was acellular and negative for any infection on microbiological stains. IgG measles antibody in CSF (10.7 mg/dl) was raised with increased CSF/Serum quotient (4.48). MRI of the brain bilateral T2/flair hyper-intensities with gyral swellings involving parieto-occipital lobes. EEG examination revealed intermittent high voltage slow wave discharges suggestive of progressive myoclonic epilepsy (Fig. 6). The above clinical history and investigations satisfied the Dyken’s criteria and the diagnosis of SSPE was made [1]. The patient was put on oral anti-convulsant for the epilepsy with supportive treatment and followed up by the neurologist. While interferon therapy can be used in such cases, our patient was not able to receive this due to financial constraints.

## Discussion

SSPE is a progressive neurodegenerative encephalopathy caused by the persistence of an aberrant mutated measles virus [2, 3]. It usually occurs 7–10 years after measles infection, but the latency varies from 1 month to 27 years [2–5]. Stringent immunization schedules in developed world has led to significant decline in the prevalence of this deadly disease to 1 per 100,000 cases [5]. Measles virus tends to affect the neurons and oligodendrocytes of brain at the time of primary systemic infection and remains quiescent for years before manifesting clinically [2]. The relationship between retinal and neurologic involvement in SSPE is not clearly understood. Ocular involvement occurs in almost 50% of SSPE cases and may antedate the onset of neurologic symptoms by several weeks or months [2]. The most characteristic ophthalmologic lesion is macular necrotizing retinitis, which spreads centrifugally to involve the posterior pole. Other protean ocular findings described with SSPE include papillitis, papilledema, optic atrophy, macular edema, serous macular detachment, or small hemorrhages within the posterior pole [6–8].

Diagnosis of SSPE is based on the Dyken’s criteria, which include two major and four minor criteria [1]. Major criteria include 1) raised anti-measles antibody titers in CSF and 2) typical or atypical clinical history. Minor criteria include 1) characteristic EEG findings that include periodic, generalized, bilaterally synchronous and symmetrical high-amplitude slow waves that recur at regular intervals of 5–15 s called Radermecker complexes, 2) CSF globulin levels greater than 20% of the total CSF protein, 3) Characteristic histopathological findings on brain biopsy,4) Molecular diagnostic test to identify wild-type measles virus mutated genome. Usually two major criteria plus one minor criterion are required, our case satisfied 2 major and 2 minor criteria and hence diagnosis of SSPE was confirmed.

Bell’s palsy (lower motor nuclear) as the first presenting neurological sign in our case is unique and never been described in literature. Residual right-sided facial nerve palsy could be appreciated at the time of appearance of retinal lesions which happened 2 months later. The characteristic hemorrhagic necrotising retinitis lesions at the macular area have been reported before [6, 7, 9]. Such retinitis lesions have been reported to be the presenting features and act as a clue to early diagnosis of this disease. The timing of appearance of these lesions have been different in most studies ranging from 6 weeks by Tripathi et al. [6] to 18 months by Shah et al. [8]. In our patient it was 8 weeks. On spectral domain optical coherence tomography (SD-OCT), there is a predominant involvement of the nuclear layers of the retina is described which tends to spread from the inner layers to the outer retinal layers. The necrosis affects first the NFL, the GCL, and then the inner nuclear layer. The outer nuclear layer becomes involved secondarily giving the entire retina disorganized moth-eaten appearance. Retinal pigment epithelium and the choroid appear uninvolved. Indeed our patient exhibited all the OCT features of SSPE [10]. FA in SSPE showed early blocked fluorescence of retinitis lesions, followed by late staining of these lesions with blocked fluorescence corresponding to the area of haemorrhages, similar to our case [7].Full blown neurological features developed about 2 months after ophthalmic lesions in the present case when she was referred to the neurologist for diagnosis and further management. Involuntary loss of bladder and bowel control occurs in these patients due to inability of the brain to control the autonomic nervous system. MRI brain of the present case showed gyral swelling involving the parieto-occipital regions, thereby explaining loss of light perception and disc pallor. Due to late diagnosis of SSPE and non-availability of isoprinosine, palliative treatment was advised by the neurologist. Supportive therapy with anti-convulsant and antispasmodic drugs and neurotonic agents were started for her.

One of the most important limitations in treatment of SSPE is difficulty in recognising early manifestations of disease, when the inflammatory changes are, possibly, still reversible. Supportive treatment including management of seizures and other complications is the mainstay of treatment. No standard treatment protocols for the treatment of SSPE have been described. Antiviral drugs and immunomodulators are used in the treatment of SSPE. Isoprinosine, interferon alfa, ribavirin, and lamivudine are the most commonly used drugs in routine clinical practice but with limited role. At present, effective measles vaccination seems to be the only beneficial and cost-effective treatment strategy to prevent this dreaded neurological disorder.

The prognosis for SSPE remains extremely guarded with a mortality of 95% of patients [2]. Average life span of a patient with SSPE is 3.8 years (range 45 days to 12 years) [2]. Timely initiation of treatment has shown to halt the progression of the disease and increase longevity of these patients but with very limited clinical improvement. Hence, prevention, in the form of measles vaccination, is the only cure for SSPE. The present case is an important learning point for us, as it reiterates the role of an ophthalmologist in early diagnosis of this deadly condition.

## Data Availability

All data generated or analyzed during this study are included in this published article.
